# AmpC β-lactamases detected in Southeast Asian *Escherichia coli* and *Klebsiella pneumoniae*

**DOI:** 10.1093/jacamr/dlae195

**Published:** 2024-11-28

**Authors:** Tamalee Roberts, Clare L Ling, Wanitda Watthanaworawit, Chanvoleak Cheav, Amphonesavanh Sengduangphachanh, Joy Silisouk, Jill Hopkins, Koukeo Phommasone, Elizabeth M Batty, Paul Turner, Elizabeth A Ashley

**Affiliations:** Lao-Oxford-Mahosot Hospital-Wellcome Trust Research Unit, Microbiology Laboratory, Mahosot Hospital, Mahosot Road, Vientiane, Lao PDR; Centre for Tropical Medicine and Global Health, Nuffield Department of Medicine, University of Oxford, Oxford, UK; Centre for Tropical Medicine and Global Health, Nuffield Department of Medicine, University of Oxford, Oxford, UK; Cambodia Oxford Medical Research Unit, Angkor Hospital for Children, Siem Reap, Cambodia; Shoklo Malaria Research Unit, Mahidol-Oxford Tropical Medicine Research Unit, Faculty of Tropical Medicine, Mahidol University, Mae Sot, Thailand; Cambodia Oxford Medical Research Unit, Angkor Hospital for Children, Siem Reap, Cambodia; Lao-Oxford-Mahosot Hospital-Wellcome Trust Research Unit, Microbiology Laboratory, Mahosot Hospital, Mahosot Road, Vientiane, Lao PDR; Lao-Oxford-Mahosot Hospital-Wellcome Trust Research Unit, Microbiology Laboratory, Mahosot Hospital, Mahosot Road, Vientiane, Lao PDR; Centre for Tropical Medicine and Global Health, Nuffield Department of Medicine, University of Oxford, Oxford, UK; Cambodia Oxford Medical Research Unit, Angkor Hospital for Children, Siem Reap, Cambodia; Lao-Oxford-Mahosot Hospital-Wellcome Trust Research Unit, Microbiology Laboratory, Mahosot Hospital, Mahosot Road, Vientiane, Lao PDR; Centre for Tropical Medicine and Global Health, Nuffield Department of Medicine, University of Oxford, Oxford, UK; Mahidol-Oxford Tropical Medicine Research Unit, Faculty of Tropical Medicine, Mahidol University, Phaya Thai, Bangkok, Thailand; Centre for Tropical Medicine and Global Health, Nuffield Department of Medicine, University of Oxford, Oxford, UK; Cambodia Oxford Medical Research Unit, Angkor Hospital for Children, Siem Reap, Cambodia; Lao-Oxford-Mahosot Hospital-Wellcome Trust Research Unit, Microbiology Laboratory, Mahosot Hospital, Mahosot Road, Vientiane, Lao PDR; Centre for Tropical Medicine and Global Health, Nuffield Department of Medicine, University of Oxford, Oxford, UK

## Abstract

**Objectives:**

AmpC β-lactamases are neglected compared with ESBL as a cause of third-generation cephalosporin (3GC) resistance in Enterobacterales in low- and middle-income countries and the burden is unknown. The aim of this study was to investigate the presence of AmpC β-lactamase-producing *Escherichia coli* and *Klebsiella pneumoniae* in clinical specimens from three clinical research laboratories in Southeast Asia.

**Methods:**

Stored clinical isolates of *E. coli* and *K. pneumoniae* resistant to ceftriaxone or ceftazidime or cefpodoxime and ESBL confirmation test negative were screened using MASTDISCS AmpC, ESBL and Carbapenemase Detection Set—D72C. Short-read WGS was performed to identify *ampC* genes.

**Results:**

Of 126 isolates collected between 2010 and 2020, 31 (24.6%) and 16 (12.7%) were phenotypically AmpC and inducible AmpC positive by MASTDISCS testing, respectively. All inducible AmpC isolates were ceftriaxone susceptible and 97.7% of AmpC/inducible AmpC isolates tested against cefoxitin were resistant. Through WGS, 17 and eight different STs were detected for the AmpC/inducible AmpC *E. coli* and *K. pneumoniae* isolates, respectively. Twelve different β-lactamase resistance genes were detected, with *bla*_CMY-2_ most commonly in AmpC-positive isolates (20/31; 64.5%; 15 chromosomal, five plasmid). All inducible AmpC-positive isolates had the *bla*_DHA-1_ gene (seven chromosomal, nine plasmid).

**Conclusions:**

Though uncommon, AmpC and inducible AmpC β-lactamases in *E. coli* and *K. pneumoniae* are an important cause of infection in Southeast Asia. With current testing methods, these infections may be going undetected, resulting in patients receiving suboptimal treatment.

## Introduction

Like ESBL-producing Enterobacterales, AmpC β-lactamase-producing bacteria have the ability to hydrolyse third-generation cephalosporins (3GCs) that are the main empirical treatment for serious community-acquired infections in Southeast Asia.^1^ Infections caused by β-lactamase-producing bacteria, including AmpC, therefore pose a threat to public health as they can lead to higher mortality rates and medical costs.^2^ Carbapenems or cefepime are usually the drugs of choice to treat serious infections caused by AmpC β-lactamase-producing bacteria. Several Enterobacterales naturally harbour an inducible chromosomal *ampC* β-lactamase gene, including *Enterobacter cloacae*, *Klebsiella aerogenes*, *Serratia marcescens*, *Citrobacter freundii*, *Providencia stuartii* and *Morganella morganii.*^3^ While *Escherichia coli* naturally carries a chromosomally mediated *ampC* gene, treatment with β-lactam antibiotics is possible due to the low-level *ampC* expression resulting from a weak promotor and a transcriptional attenuator preceding the *ampC* gene.^4^ However, mutations in the promotor or attenuator region may result in hyperexpression of *ampC* and strains may become resistant to some antibiotics including 3GCs.^4^ While acquired *ampC* is usually fully expressed constitutively, some plasmid-carried *ampC* genes such as DHA-1 are inducible by β-lactams but with expression regulated similarly to that of inducible chromosomal *ampC* genes.^5^ Common *ampC* genes previously found in animals and humans from the Asia-Pacific region are the CIT gene group including *bla*_CMY-2_, the DHA group including *bla*_DHA-1_, the ACT/MIR group and the FOX group.^2,6^ Various *ampC* genes can translocate between chromosomes and plasmids, resulting in rapid spread through the environment that can lead to dissemination of resistance and global clonal spread.^1^ The spread of plasmids and acquisition of *ampC* β-lactamase genes by *E. coli* and *Klebsiella* spp. can cause hospital outbreaks, which can have a huge financial impact on the healthcare setting and patient treatment. Surveillance for the existence of AmpC β-lactamases is important for clinical care and infection control.

AmpC β-lactamases appear to be less prevalent than ESBLs; however, accurate prevalence data are lacking due to an absence of testing. AmpC β-lactamases have previously been described from humans in Thailand and Cambodia but studies are limited,^7,8^ and there are no data on AmpC β-lactamases in the Lao People’s Democratic Republic (Laos). AmpC β-lactamases are typically resistant to cefoxitin and this can been used as a screening tool for the detection of AmpC β-lactamase producers.^1^ However, isolates with an ACC type enzyme may appear to be cefoxitin susceptible and missed by cefoxitin screening, though these enzymes appear to be rare. Organisms producing a carbapenemase can also mimic those with an AmpC β-lactamase with cefoxitin inactivation so carbapenemase susceptibility should also be determined.^1^

The aim of this study was to investigate the presence of AmpC β-lactamase-producing *E. coli* and *Klebsiella pneumoniae* from clinical specimens from three clinical research laboratories in Southeast Asia (Cambodia, Laos and Thailand) phenotypically using the MASTDISCS AmpC, ESBL and Carbapenemase Detection Set (MAST, D72C; referred to as MASTDISCS). The MASTDISCS kit consists of six different antibiotic discs: A, cefpodoxime; B, cefpodoxime plus ESBL inhibitor; C, cefpodoxime + AmpC inhibitor; D, cefpodoxime plus ESBL inhibitor plus AmpC inhibitor; E, cefpodoxime plus ESBL inhibitor plus AmpC inducer; and F, penem antibiotic. This kit enables the detection of AmpC and inducible AmpC phenotypes, as well as ESBL and carbapenemase resistance, and may give an equivocal result. In addition, WGS was employed to investigate genetic determinants of AmpC phenotypes. The results from this study provide much-needed information on AmpC β-lactamase epidemiology in the region and will help guide local infection control, surveillance and treatment guidelines.

## Materials and methods

### Study sites

Clinical isolates from three clinical research sites in Southeast Asia were included in the study: the Cambodia Oxford Medical Research Unit (COMRU), Siem Reap, Cambodia, which provides the diagnostic microbiology service at Angkor Hospital for Children, an ∼100-bed non-governmental paediatric hospital;^9^ the Lao-Oxford-Mahosot Hospital-Wellcome Trust Research Unit (LOMWRU), Vientiane, Laos, which partially supports the diagnostic microbiology service at Mahosot Hospital, an ∼400-bed government hospital providing primary, secondary and tertiary care and admitting ∼2000 patients/month;^10^ and the Shoklo Malaria Research Unit (SMRU), Mae Sot, Thailand, which provides healthcare to the marginalized populations living on both sides of the Thailand–Myanmar border in Tak province.^11^

### Isolate selection

Stored clinical isolates previously identified as *E. coli* or *K. pneumoniae* by conventional identification methods and that were resistant to ceftriaxone or ceftazidime or cefpodoxime (Oxoid) by routine antimicrobial susceptibility testing (AST) but were ESBL confirmation test negative [by VITEK 2 or by the double-disc method: cefotaxime ± clavulanate and ceftazidime ± clavulanate (BD)] were selected. For COMRU and SMRU, all available isolates were selected, and for LOMWRU a random subset of isolates was selected. Identification methods included biochemical testing, API (bioMérieux) or MALDI-TOF MS (VITEK MS, bioMérieux). AST was performed using disc diffusion (Oxoid, COMRU until May 2019, LOMWRU and SMRU) or VITEK 2 (GN84 AST cards, bioMérieux; COMRU from May 2019) and results were interpreted following the current guidelines at the time of testing: CLSI (COMRU, SMRU and LOMWRU until 2018) or EUCAST (LOMWRU from 2018). In addition, cefoxitin AST was performed retrospectively at COMRU following EUCAST guidelines for isolates with WGS results. Isolates from patients were separated into episodes, with one patient episode defined as the same patient and organism isolated within 14 days.

### AmpC screening

Selected isolates were phenotypically tested using the MASTDISCS AmpC, ESBL and Carbapenemase Detection Set—D72C (referred to as MASTDISCS) according to the manufacturer’s guidelines (Mast Group Ltd). In brief, 0.5 McFarland saline suspensions, made using fresh subcultures, were spread onto Mueller–Hinton agar plates. Room temperature MASTDISCS were placed on the plates in alphabetical order and plates were then incubated in air at 35°C–37°C for 18–24 h. The zones of inhibition were measured to the nearest whole millimetre and any microcolonies present in disc F zones of inhibition were recorded. The Excel sheet calculator provided by the manufacturer was used to determine the resistance mechanisms (D72C-AmpC_ESBL_Carba_Calculator_v4.0.xlsx; available from www.mast-group.com).

### WGS

WGS was performed on 77 isolates: all isolates identified as AmpC, inducible AmpC or AmpC plus ESBL positive by MASTDISCS; and 25 isolates from other MASTDISCS result groups that had previously been sequenced for other purposes. The remaining isolates were not sequenced due to financial constraints. DNA was extracted from isolates using the Wizard Genomic DNA purification kit (Promega) at SMRU and COMRU, the GeneJET Genomic DNA Purification kit (Thermo Scientific™) at LOMWRU and the Ultraclean DNeasy kit (QIAGEN) at COMRU. WGS was performed at COMRU for 68 isolates using Illumina library preparation kits and the Illumina iSEQ100 platform, yielding 150 bp paired-end reads, and nine were sequenced as part of the Real-time Tracking of Neglected Bacterial Infectious Diseases Resistance Patterns Asia (TuNDRA) study using an Illumina HiSeq platform (unpublished data).

The Bactopia pipeline (v2.1.1) was used to assemble and analyse sequences with default parameters.^12^ Only sequences meeting minimum quality thresholds were included in the analysis (≥20× coverage, ≥Q12 minimum per-read mean quality score, minimum mean read length of ≥49 bp and ≤500 total contigs; the Bactopia quality thresholds for ‘bronze’ level). Analysis tools executed via Bactopia included MLST (v2.22.0), ECTyper (v.1.0.0) and Kleborate (v2.1.0) for *in silico* prediction of STs, *E. coli* serotypes and *Klebsiella* speciation, respectively.^13–15^ Unresolved STs were confirmed with the online EnteroBase tool (v1.2.0).^16^ Chromosomal and plasmid contigs were identified using Platon (v1.6).^17,18^ AMRFinderPlus (v3.12.8 with database v2023–04–28) was used to identify antimicrobial resistance (AMR) genes. All identified genes had ≥95% coverage and ≥95% identity with their closest matched reference genes, unless otherwise stated. Assemblies containing gene hits with ≥95% identity, but ≤95% coverage were further investigated by performing BLAST searches with the relevant reference genes obtained from the National Library of Medicine (National Institutes of Health; U.S. Department of Health and Human Services).^19^ Snippy (v4.6.0), with reference genomes *E. coli* str. K12 substr. MG1655 (U00096.3) and *K. pneumoniae* subsp. *pneumoniae* HS11286 (CP003200.1), was used to produce variant calls, followed by snippy-core to produce core-SNP alignments.^20^ Phylogenetic trees were created from the core-SNP alignments with IQ-Tree (v1.6.12) and visualized/annotated using ggtree via R (v4.2.1).^21,22^ Clinker v0.0.03 (via the CAGECAT webserver version 1.0 [https://cagecat.bioinformatics.nl/]) was used to visualize flanking regions of *bla*_DHA-1_, *bla*_CMY-2_ and *bla*_CMY-42_ genes for contigs greater than 5 kb in length, using the default settings.

### Quality control

For identification and AST, internal quality control (IQC) was completed for all media and antibiotics following the laboratories’ IQC routine protocols. For MASTDISCS, *E. coli* ATCC 25922 (negative control), known ESBL-negative and known ESBL-positive clinical isolates were used at all sites except COMRU, where *K. pneumoniae* ATCC 700603 was used as the ESBL-positive control and *K. pneumoniae* ATCC BAA-1705 as the carbapenemase-producer control. There was no AmpC control available at any of the sites. All sites participate in external quality assurance (EQA) programmes: COMRU participates in the Pacific Pathology Training Centre Microbiology Quality Assurance Programme; LOMWRU participates in the UK National EQA Scheme; and SMRU participates in the Thailand Clinical Microbiology Proficiency Testing Scheme, Division of Proficiency Testing, Department of Medical Science, Ministry of Public Health. Results are reported following the MICRO Checklist (File S1, available as Supplementary data at *JAC-AMR* Online).^23^ During the isolate selection period of this study, no sites had ISO accreditation; however, COMRU was accredited on the 23 November 2023 according to ISO 15189:2012 and ISO 15190:2020 standards (accreditation no. 4340/67).

### Data analysis

All data were entered into Microsoft Excel (Richmond, WA, USA). Analysis was performed using Stata version 16.1 (StataCorp, College Station, TX, USA).

## Results

**Figure 1.**
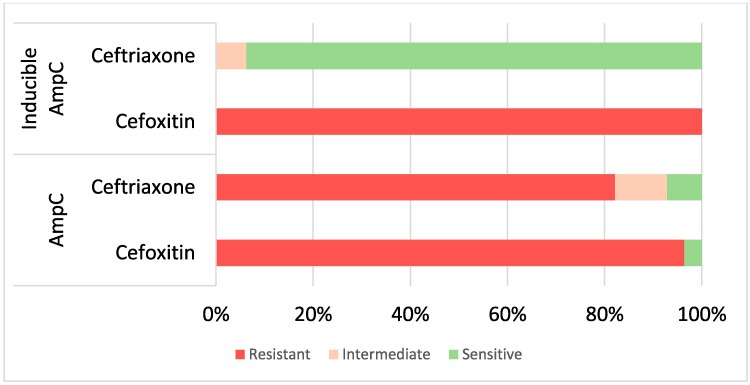
Cefoxitin and ceftriaxone AST interpretations for isolates with phenotypic MASTDISCS D72C results of AmpC and inducible AmpC (44 isolates; four isolates excluded as they were not tested with cefoxitin).

WGS revealed one of the selected *K. pneumoniae* isolates was actually *Klebsiella quasipneumoniae* subsp. *similipneumoniae* and it was excluded from the analysis. Although *K. quasipneumoniae* subsp. *similipneumoniae* can cause infections similar to *K. pneumoniae*, it was excluded as it was not one of our target organisms and other *Klebsiella* spp. were not included in the study. Although 130 isolates were tested by MASTDISCS, only the first *E. coli* and/or first *K. pneumoniae* isolate from each illness episode were included in the analysis, resulting in the exclusion of four isolates from three patients. One patient had an *E. coli* and a *K. pneumoniae* isolated from the same episode/specimen, and both were included in the analysis. A total of 126 isolates (110 *E. coli* and 16 *K. pneumoniae*) from 125 illness episodes and from 121 patients from COMRU (*n* = 76), LOMWRU (*n* = 42) and SMRU (*n* = 8) collected between 2010 and 2020 were included in the study (Table S1). For the isolate selection, there were 100/126 (79.4%) isolates resistant to ceftriaxone, 93/101 (92.1%) isolates resistant to ceftazidime and 77/83 (92.8%) isolates resistant to cefoxitin. Isolates were from urine (*n* = 72), blood culture (*n* = 29), pus (*n* = 20) and other sites (*n* = 5). The MASTDISCS results identified 47/126 (37.3%) AmpC-positive isolates including 31/126 (24.6%) AmpC positive and 16 (12.7%) inducible AmpC. There was: one (0.8%) inducible AmpC, suspected carbapenemase or suspected ESBL/AmpC with porin loss (referred to as Undetermined Group 1); 41 (32.5%) suspected carbapenemase or suspected ESBL/AmpC with porin loss (referred to as Undetermined Group 2); four (3.2%) AmpC and ESBL positive; three (2.4%) ESBL positive; eight (6.3%) AmpC and ESBL negative; and 22 (17.5%) equivocal (Table S2). There were no isolates positive for both AmpC and inducible AmpC. From AST results, 25/31 (80.6%) AmpC-positive isolates were phenotypically ceftriaxone resistant, 15/16 (93.8%) inducible AmpC isolates were ceftriaxone susceptible and 1/16 (6.3%) was ceftriaxone susceptible, increased exposure. All tested AmpC/inducible AmpC-positive isolates were resistant to cefoxitin except one (43/44; 97.7%) (Figure 1 and Table S2). Fifteen controls were tested (seven ESBL positive, seven ESBL negative and one carbapenemase producer) and all gave expected MASTDISCS results. For COMRU, between 2012 and 2020 the prevalence of AmpC/inducible AmpC was 1.9% for *E. coli* and 0.7% for *K. pneumoniae* (17/879 and 2/273 episodes, respectively). As selected isolates were tested for LOMWRU, the prevalence during the study period is not known; however, in 2023, 3.3% (20/600) of *E. coli* and 2% (5/254) of *K. pneumoniae* isolates were AmpC or inducible AmpC positive. For SMRU, between 2016 and 2020 the prevalence of AmpC *E. coli* was 0.4% (3/854).

### Sequencing results

**Figure 2.**
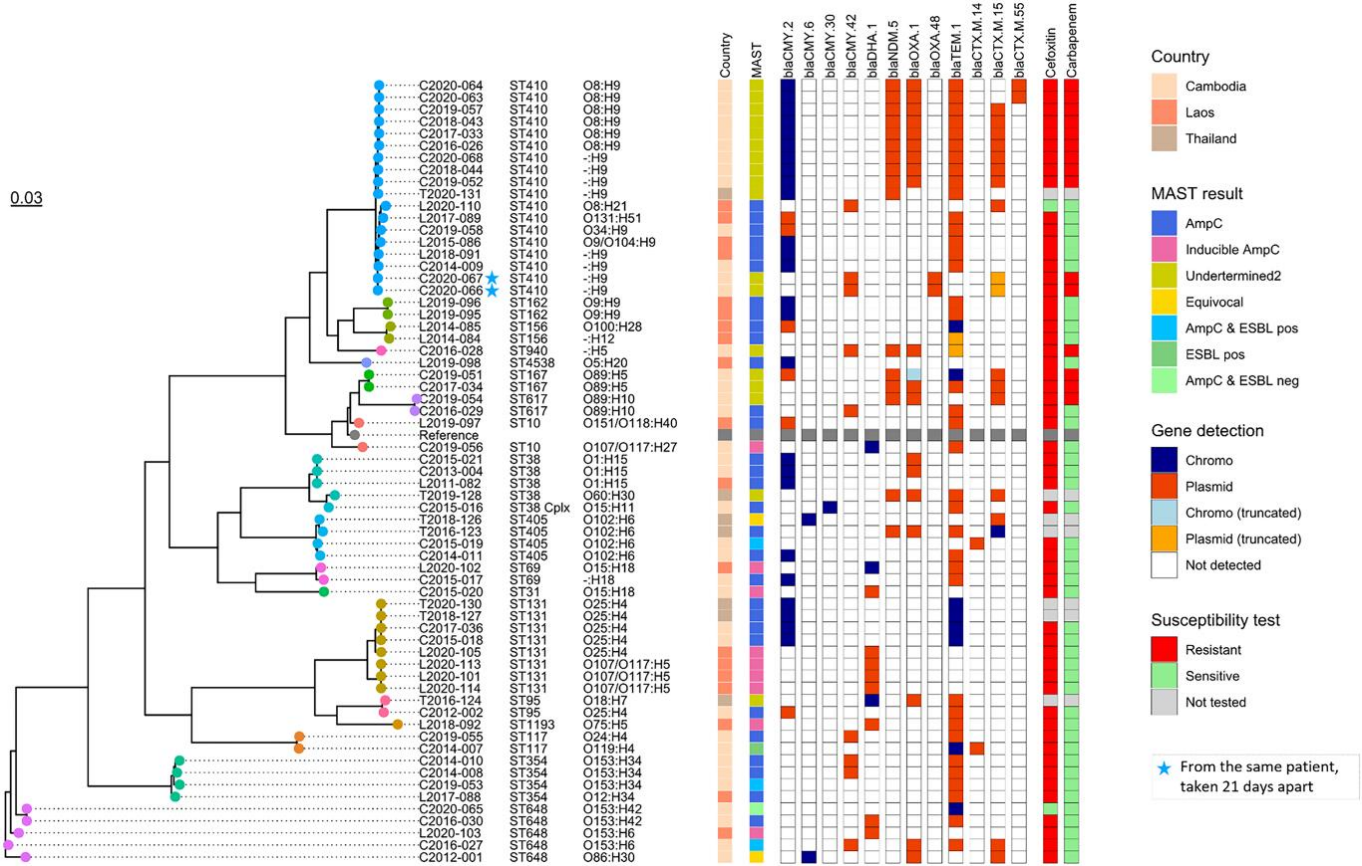
Phylogenetic tree of 64 *E. coli* isolates showing ST, serotype, country of isolate origin, MASTDISCS result, resistance genes detected and if chromosomal or plasmid, susceptibility to cefoxitin and a carbapenem antibiotic if at least one tested. Reference strain is *E. coli* str. K12 (U00096.3).

**Figure 3.**
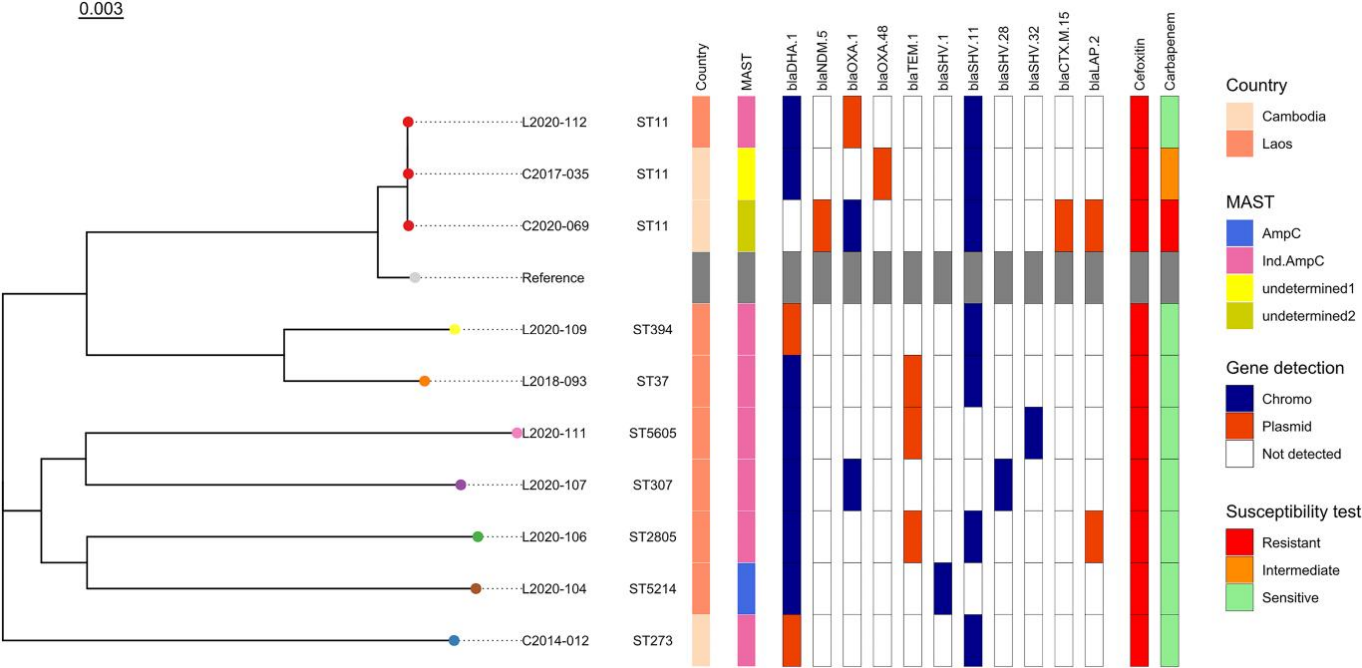
Phylogenetic tree of 10 *K. pneumoniae* isolates showing ST, country of isolate origin, MAST disc result, resistance genes detected and if chromosomal or plasmid, susceptibility to cefoxitin and a carbapenem antibiotic if at least one tested. Reference strain is *K. pneumoniae* subsp. *pneumoniae* HS11286 (CP003200.1).

Excluding the *K. quasipneumoniae* isolate, a total of 74 isolates were sequenced (64 *E. coli* and 10 *K. pneumoniae*; 41 from COMRU, 26 from LOMWRU and seven from SMRU) including all the detected AmpC-, inducible AmpC- and AmpC plus ESBL-positive isolates. STs, serotypes and identified β-lactamase genes are provided in Table 1, Tables S3–S6, Figures 2–3 and Figures S1–S2.

**Table 1.** Combined sequencing results for a subset of 74 *E. coli* and *K. pneumoniae* isolates and phenotypic MASTDISC D72C results

Genes	MASTDISCS results (T/C/P)								Total
	AmpC	Inducible AmpC	AmpC and ESBL positive	ESBL positive	Equivocal	Undetermined Group 1	Undetermined Group 2	AmpC & ESBL negative	
	*n* = 31	*n *= 16	*n* = 3	*n* = 1	*n* = 2	*n *= 1	*n *= 19	*n* = 1	*n* = 76
*bla*_CMY-2_	20/15/5	0	0	0	0	0	11/10/1	0	31/25/6
*bla*_CMY-6_	0	0	0	0	2/2/0	0	0	0	2/2/0
*bla*_CMY-30_	1/1/0	0	0	0	0	0	0	0	1/1/0
*bla*_CMY-42_	5/0/5	0	1/0/1	0	0	0	3/0/3	0	9/0/9
*bla*_DHA-1_	2/1/1	16/7/9	0	0	0	1/1/0	1/1/0	0	20/10/10
*bla*_NDM-5_	1/0/1	0	0	0	0	0	16/0/16	0	17/0/17
*bla*_OXA-1_	3/0/3	2/1/1	1/0/1	0	1/0/1	0	15/2^a^/14	0	23/3/20
*bla*_OXA-48_	0	0	0	0	0	1/0/1	2/0/2	0	3/0/3
*bla*_TEM-1_	25/5/20^a^	6/0/6	2/0/2	1/1/0	0	0	15/1/14^a^	1/1/0	50/8/42
*bla*_SVH-1_	1/1/0	0	0	0	0	0	0	0	1/1/0
*bla*_SVH-11_	0	5/5/0	0	0	0	1/1/0	1/1/0	0	7/7/0
*bla*_SVH-28_	0	1/1/0	0	0	0	0	0	0	1/1/0
*bla*_SVH-32_	0	1/1/0	0	0	0	0	0	0	1/1/0
*bla*_CTX-M-14_	0	0	1/0/1	1/0/1	0	0	0	0	2/0/2
*bla*_CTX-M-15_	2/1/1	0	1/0/1	0	2/0/2	0	14/0/14^b^	0	19/1/18
*bla*_CTX-M-55_	0	0	0	0	0	0	2/0/2	0	2/0/2
*bla*_LAP-2_	0	1/0/1	0	0	0	0	1/0/1	0	2/0/2

*n*, number of isolates with a MASTDISC result; T, total amount with gene; C, number found on the chromosome; P, number found on a plasmid; Undetermined Group 1, inducible AmpC suspected carbapenemase or suspected ESBL/AmpC with porin loss; Undetermined Group 2, suspected carbapenemase or suspected ESBL/AmpC with porin loss. Only genes with ≥95% coverage and ≥95% identification with a reference gene are included, unless otherwise stated.

^a^Truncated gene detected for one isolate.

^b^Truncated gene detected for two isolates.

A chromosomal class C β*-*lactamase *bla*_EC_ gene was detected for all *E. coli* (15 *bla*_EC-5_, 14 *bla*_EC-8_, 25 *bla*_EC-15_, five *bla*_EC-18_ and five *bla*_EC-19_ including one isolate where two truncated genes with ≥95% identity, but only 72% coverage with *bla*_EC-5_ and *bla*_EC-15_ reference genes were detected). For the 31 phenotypically AmpC-positive isolates, 26 had a *bla*_CMY_ gene: 20 (64.5%) had *bla*_CMY-2_ genes (15 chromosomal, five plasmid); five (16.1%) had *bla*_CMY-42_ genes (all plasmid) and one had a *bla*_CMY-30_ gene (this gene was split across two chromosomal contigs). Of the five AmpC-positive isolates with no detected *bla*_CMY_ gene, two (6.5%) had *bla*_DHA-1_ genes (one chromosomal, one plasmid) and no additional class C β*-*lactamase gene was detected for three (9.7%). Class C β*-*lactamase *bla*_CMY_ genes were also detected in isolates with other MASTDISCS results: AmpC/ESBL positive (*n* = 1/3), equivocal (*n* = 2/2) and Undetermined Group 2 (*n* = 14/19) but they were not detected in inducible AmpC isolates (*n* = 16), AmpC/ESBL negative (*n* = 1), ESBL-positive (*n* = 1) or Undetermined Group 1 isolates (*n* = 1). The phenotypically inducible AmpC-positive isolates all had the class C β*-*lactamase *bla*_DHA-1_ gene (16; seven chromosomal and nine plasmid). Isolates with other MASTDISCS results carrying this gene were Undetermined Group 1 (*n* = 1/1) and Undetermined Group 2 result (*n* = 1/19). Using clinker, common flanking gene clusters were identified for *bla*_DHA-1_, *bla*_CMY-2_ and *bla*_CMY-42_ genes (Figures S3–S4c). Isolates with *ampC* genes determined to be chromosomal and those determined as plasmid-borne tended to cluster separately, with different flanking genes. With the exception of plasmid *bla*_DHA-1_ genes, the *ampC* genes tended to be close to the start or end of the contigs, limiting information on both flanking regions. For *bla*_DHA-1_, a cluster of eight flanking genes were shared in 18 out of 19 isolates across *E. coli* and *K. pneumoniae.* The cluster included genes for AmpR (a transcriptional regulator), phage shock proteins and envelope stress response membrane proteins. Additionally, the sulphonamide resistance gene *sul1* was found for eight isolates with plasmid *bla*_DHA-1_. The flanking genes for *bla*_CMY-2_ and *bla*_CMY-42_ differed from those found for *bla*_DHA-1_, with most being uncharacterized proteins. For plasmid *bla*_CMY-42_, eight out of the nine isolates visualized with clinker had multiple flanking genes in common. Chromosomal *bla*_CMY-2_ isolates were similar to each other, but differed to the plasmid *bla*_CMY-2_ isolates, where greater variation in flanking regions was observed.

A total of 19 different STs were detected for *E. coli*, of which the most common were ST410 (18/64; 28.1%), ST131 (8/64; 12.5%) and ST648 (5/64; 7.8%). For the AmpC-positive isolates, 15 different STs were detected, most commonly ST410 (6/30; 20.0%) and ST131 (4/30; 13.3%). Inducible AmpC-positive isolates had six different STs, most commonly ST131 (4/9; 44.3%) and no ST410. For *K. pneumoniae*, eight different STs were detected, including seven different STs for the phenotypically inducible AmpC isolates (*n* = 7). ST11 was the most common *K. pneumoniae* ST (3/10; 30%).

Other β-lactamase/carbapenemase genes detected from the combined 47 phenotypic AmpC-positive and inducible AmpC isolates included 31/47 (66%) *bla*_TEM-1_, 5/47 (10.6%) *bla*_OXA-1_, 5/47 (10.6%) *bla*_SHV-11_, 1/47 (2.1%) *bla*_SHV-28_, 2/47 (4.3%) *bla*_CTX-M-15_, 1/47 (2.1%) *bla*_LAP-2_ and 1/47 (2.1%) *bla*_NDM-5_. For the two *E. coli* isolates with equivocal results from the MASTDISCS, both had *bla*_CMY-6_ and *bla*_CTX-M-15_ genes and one isolate also had *bla*_OXA-1_. Among the 23 isolates harbouring a *bla*_OXA-1_ gene, 14 showed resistance to meropenem and/or imipenem, six were susceptible and three were not tested with a carbapenem antibiotic. For the isolates with *bla*_OXA-48_, two out of three (66.7%) were susceptible to both meropenem and imipenem, and one was meropenem intermediate (33.3%, imipenem was not tested). Of the 17 isolates harbouring *bla*_NDM-5_, 16 gave Undetermined Group 2 MASTDISCS results, with 14 resistant to meropenem and/or imipenem and two not tested; one isolate gave an AmpC-positive MASTDISCS result for which no carbapenem AST result was available. The six phenotypically AmpC isolates that were ceftriaxone susceptible, intermediate or susceptible, increased exposure, harboured a variety of genes and all six did not have the same genes.

## Discussion

This study describes AmpC β-lactamase production from *E. coli* and *K. pneumoniae* isolates from three clinical laboratories in Southeast Asia. The study highlights that 37% of isolates that were ESBL confirmation test negative were phenotypically AmpC or inducible AmpC positive using the MASTDISCS. All of the inducible AmpC isolates were phenotypically ceftriaxone susceptible or susceptible, increased exposure. This can have an impact on patient care if treatment is based on susceptibility results, without further testing for AmpC production, as failure on treatment has been described in this situation,^3^ and it is generally recommended that treatment with 3GCs should be avoided, regardless of ceftriaxone susceptibility results. Of the 44 AmpC and inducible AmpC isolates that were tested against cefoxitin, 43 (97.7%) were cefoxitin resistant. This shows the effectiveness of cefoxitin as a screening tool for AmpC β-lactamases. Cefoxitin screening is currently part of EUCAST guidelines, with reported high sensitivity but poor specificity, but not CLSI guidelines. If laboratories are not screening using cefoxitin and are reliant on ceftriaxone testing, they may be missing AmpC- and/or inducible AmpC-positive isolates. The prevalence of AmpC or inducible AmpC *E. coli* during the study period or subsequent years ranged for the different sites from 0.4% to 3.3% (not including additional AmpC production from other MASTDISCS groups), indicating the need for local prevalence determination.

Despite the small sample size, there were a diverse number of STs detected. The predominant STs for *E. coli* were ST410 and ST131, and the predominant ST for *K. pneumoniae* was ST11. ST410 and ST131 have recently been categorized as ‘high-risk’ *E. coli* clones internationally,^24,25^ with ST410 identified in Cambodia, Vietnam and Thailand previously.^26^ While *bla*_CTX-M-15_-encoding genes are associated with ST131,^25^ there was only one isolate with this gene (ESBL positive and the *bla*_CTX-M-15_ located on the chromosome) while the other eight isolates had a variety of *bla*_CMY-2_, *bla*_TEM-1_ and *bla*_DHA-1_ genes. ST11 has been associated with carbapenem-resistant *K. pneumoniae* and has become the dominant clone in many countries.^27^ The three ST11 isolates in this study all had common carbapenemase-encoding genes (*bla*_NDM_ and *bla*_OXA_); however, only the isolate that had a *bla*_NDM-5_ gene was meropenem resistant.

The most common AmpC genes detected in this study were *bla*_CMY-2_ and *bla*_DHA-1_, which is similar to other studies in the region.^2,6^ These genes were located on both chromosomes and plasmids, whereas the less common *bla*_CMY-42_ was only located on the plasmid. Previously, *bla*_DHA-1_ has most frequently been reported on plasmids, so finding it on the chromosome could be confirmed by long-read sequencing. All inducible AmpCs carried *bla*_DHA-1_. The *bla*_EC_ gene was present in all the *E. coli* isolates; however, only three phenotypically AmpC isolates had this Class C gene without a *bla*_CMY_ or *bla*_DHA-1_ gene. These three isolates were also phenotypically susceptible or intermediate to ceftriaxone by disc diffusion. While a large number of *bla*_EC_ variants have been described, only some are expressed to a sufficient extent to cause antibiotic resistance.^28^ Common gene cluster patterns were identified in isolates containing *ampC* genes, with distinct patterns of flanking genes observed for chromosomal versus plasmid-borne genes, and for *bla*_DHA-1_ versus *bla*_CMY-2_/*bla*_CMY-42_ genes. Although the analysis was limited by short-read sequencing data, a shared group of eight genes was identified for both plasmid and chromosomal *bla*_DHA-1_-harbouring isolates, indicating that plasmid integration of *bla*_DHA-1_ into the chromosome may have occurred as a single event. In contrast, *bla*_CMY-2_ was associated with many different flanking genes, suggesting no single mobile genetic element is responsible for the transmission of *bla*_CMY-2_ in these isolates. Additionally, an AMR gene was found flanking plasmid *bla*_DHA-1_ genes, which may facilitate transmission of multidrug resistance.

Of the sequenced isolates, excluding Undetermined Groups 1 and 2, 1/54 had a *bla*_NDM-5_ gene and 7/54 had a *bla*_OXA-1_ gene. All *bla*_NDM-5_ and *bla*_OXA-48_ genes in this study were located on plasmids, whereas the *bla*_OXA-1_ genes were found on both plasmids and chromosomes. There were two *K. pneumoniae* isolates with *bla*_LAP-2_ genes, one phenotypically inducible AmpC (ST2805 with genes *bla*_DHA-1_, *bla*_TEM-1_, *bla*_SVH-11_) and one suspected carbapenemase or suspected ESBL/AmpC with porin loss (ST11 with genes *bla*_SVH-11_, *bla*_CTX-M-15_, *bla*_NDM-5_ and *bla*_OXA-1_). *bla*_LAP_ is a narrow-spectrum Class A β-lactamase but reports are limited.^29^ Overall, the finding of multiple resistance genes located on both chromosomes and plasmids has implications for infection control, with transmission dynamics and dissemination of resistant strains difficult to predict.

While the MASTDISCS test was beneficial for the identification of most categories, the two categories of suspected carbapenemase or suspected ESBL/AmpC with porin loss and inducible AmpC suspected carbapenemase or suspected ESBL/AmpC with porin loss can make interpretation of results difficult when relying solely on this test. However, we still believe this test is beneficial for determining resistance mechanisms in clinical and resource-limited settings where WGS is not available.^30^ From the sequencing results, it is clear that there are many different resistance gene classes found in these isolates and that they are difficult to categorize phenotypically.

There were several limitations to this study. Firstly, different AST methods were used between and within sites during the study period. With yearly AST guideline updates from CLSI and EUCAST, interpretation of zone sizes may change over time and isolates that were susceptible may become resistant or vice versa. Secondly, although all phenotypically AmpC and inducible AmpC isolates were sequenced, not all isolates from the other phenotypic groups were sequenced due to financial constraints and therefore not all STs and resistance genes will have been captured for these groups. And lastly, the correct control strains were not available at all the sites for the MASTDISCS testing so thorough quality control was not completed. If MASTDISCS were to be included for routine testing, the appropriate quality control strains would be needed.

### Conclusions

Our findings confirm that AmpC β-lactamase-producing *E. coli* and *K. pneumoniae* are an important, although uncommon, cause of infection in patients presenting to hospitals in Cambodia, Laos and Thailand and are less likely to respond to ceftriaxone, the main first-line treatment for sepsis. These results highlight that with current AST, inducible AmpC β-lactamases may be going undetected if routine screening is not performed. Cefoxitin-resistant, ESBL-negative isolates should be screened for AmpC β-lactamases.

## Supplementary Material

dlae195_Supplementary_Data

## Data Availability

The metadata supporting the conclusions of this article with de-identified data and data dictionary, and the additional files are available in the Figshare repository https://doi.org/10.6084/m9.figshare.26087785.v3. The sequences were deposited in the European Nucleotide Archive (ENA) at EMBL-EBI under project accession number PRJEB76280 (will be made public on acceptance, accession numbers in metadata).

## Supplementary data

File S1, Figures S1–S4 and Tables S1–S6 are available as Supplementary data at *JAC-AMR* Online.

## References

[1] Jacoby GA . AmpC β-lactamases. Clin Microbiol Rev 2009; 22: 161–82. 10.1128/CMR.00036-08 19136439 PMC2620637

[2] Sheng WH, Badal RE, Hsueh PR et al Distribution of extended-spectrum β-lactamases, AmpC β-lactamases, and carbapenemases among Enterobacteriaceae isolates causing intra-abdominal infections in the Asia-Pacific region: results of the Study for Monitoring Antimicrobial Resistance Trends (SMART). Antimicrob Agents Chemother 2013; 57: 2981–8. 10.1128/AAC.00971-12 23587958 PMC3697370

[3] Tamma PD, Doi Y, Bonomo RA et al A primer on AmpC β-lactamases: necessary knowledge for an increasingly multidrug-resistant world. Clin Infect Dis 2019; 69: 1446–55. 10.1093/cid/ciz173 30838380 PMC6763639

[4] Coolen JPM, den Drijver EPM, Kluytmans JAJW et al Development of an algorithm to discriminate between plasmid- and chromosomal-mediated AmpC β-lactamase production in *Escherichia coli* by elaborate phenotypic and genotypic characterization. J Antimicrob Chemother 2019; 74: 3481–8. 10.1093/jac/dkz362 31504559 PMC7183348

[5] Realegeno S, Ward K, Garner OB et al Deceiving phenotypic susceptibility results on a *Klebsiella pneumoniae* blood isolate carrying plasmid-mediated AmpC gene *bla*_DHA-1_. Front Cell Infect Microbiol 2021; 11: 561880. 10.3389/fcimb.2021.561880 33791229 PMC8006929

[6] Nguyen DP, Nguyen TAD, Le TH et al Dissemination of extended-spectrum β-lactamase- and AmpC β-lactamase-producing *Escherichia coli* within the food distribution system of Ho Chi Minh City, Vietnam. Biomed Res Int 2016; 2016: 8182096. 10.1155/2016/8182096 26989692 PMC4773546

[7] Atterby C, Osbjer K, Tepper V et al Carriage of carbapenemase- and extended-spectrum cephalosporinase-producing *Escherichia coli* and *Klebsiella pneumoniae* in humans and livestock in rural Cambodia; gender and age differences and detection of *bla*_OXA-48_ in humans. Zoonoses Public Health 2019; 66: 603–17. 10.1111/zph.12612 31264805 PMC6852310

[8] Singtohin S, Chanawong A, Lulitanond A et al CMY-2, CMY-8b, and DHA-1 plasmid-mediated AmpC β-lactamases among clinical isolates of *Escherichia coli* and *Klebsiella pneumoniae* from a university hospital, Thailand. Diagn Microbiol Infect Dis 2010; 68: 271–7. 10.1016/j.diagmicrobio.2010.06.014 20851550

[9] Fox-Lewis A, Takata J, Miliya T et al Antimicrobial resistance in invasive bacterial infections in hospitalized children, Cambodia, 2007–2016. Emerg Infect Dis 2018; 24: 841–51. 10.3201/eid2405.171830 29664370 PMC5938766

[10] Dubot-Peres A, Mayxay M, Phetsouvanh R et al Management of central nervous system infections, Vientiane, Laos, 2003–2011. Emerg Infect Dis 2019; 25: 898–910. 10.3201/eid2505.180914 31002063 PMC6478220

[11] SMRU . Shoklo Malaria Research Unit. https://www.shoklo-unit.com/.

[12] Petit RA 3rd, Read TD. Bactopia: a flexible pipeline for complete analysis of bacterial genomes. mSystems 2020; 5: e00190-20. 10.1128/mSystems.00190-20 32753501 PMC7406220

[13] Jolley KA, Bray JE, Maiden MCJ. Open-access bacterial population genomics: BIGSdb software, the PubMLST.org website and their applications. Wellcome Open Res 2018; 3: 124. 10.12688/wellcomeopenres.14826.1 30345391 PMC6192448

[14] Lam MMC, Wick RR, Watts SC et al A genomic surveillance framework and genotyping tool for *Klebsiella pneumoniae* and its related species complex. Nat Commun 2021; 12: 4188. 10.1038/s41467-021-24448-3 34234121 PMC8263825

[15] Laing C, Bessonov K, Sung S et al ECTyper—in silico prediction of *Escherichia coli* serotype. 2021. https://github.com/phac-nml/ecoli_serotyping. 10.1099/mgen.0.000728 PMC8767331 34860150

[16] Zhou Z, Alikhan NF, Mohamed K et al The EnteroBase user’s guide, with case studies on *Salmonella* transmissions, *Yersinia pestis* phylogeny, and *Escherichia* core genomic diversity. Genome Res 2020; 30: 138–52. 10.1101/gr.251678.119 31809257 PMC6961584

[17] Schwengers O, Barth P, Falgenhauer L et al Platon: identification and characterization of bacterial plasmid contigs in short-read draft assemblies exploiting protein sequence-based replicon distribution scores. Microb Genom 2020; 6: mgen000398. 10.1099/mgen.0.000398 32579097 PMC7660248

[18] Robertson J, Nash JHE. MOB-suite: software tools for clustering, reconstruction and typing of plasmids from draft assemblies. Microb Genom 2018; 4: e000206. 10.1099/mgen.0.000206 30052170 PMC6159552

[19] Altschul SF, Gish W, Miller W et al Basic local alignment search tool. J Mol Biol 1990; 215: 403–10. 10.1016/S0022-2836(05)80360-2 2231712

[20] Seeman T. Snippy. 2018. https://github.com/tseemann/snippy.

[21] Yu G . Using ggtree to visualize data on tree-like structures. Curr Protoc Bioinformatics 2020; 69: e96. 10.1002/cpbi.96 32162851

[22] Nguyen LT, Schmidt HA, von Haeseler A et al IQ-TREE: a fast and effective stochastic algorithm for estimating maximum-likelihood phylogenies. Mol Biol Evol 2015; 32: 268–74. 10.1093/molbev/msu300 25371430 PMC4271533

[23] Turner P, Fox-Lewis A, Shrestha P et al Microbiology investigation criteria for reporting objectively (MICRO): a framework for the reporting and interpretation of clinical microbiology data. BMC Med 2019; 17: 70. 10.1186/s12916-019-1301-1 30922309 PMC6440102

[24] Roer L, Overballe-Petersen S, Hansen F et al *Escherichia coli* sequence type 410 is causing new international high-risk clones. mSphere 2018; 3: e00337-18. 10.1128/mSphere.00337-18 30021879 PMC6052333

[25] Carvalho I, Carvalho JA, Martínez-Álvarez S et al Characterization of ESBL-producing *Escherichia coli* and *Klebsiella pneumoniae* isolated from clinical samples in a Northern Portuguese hospital: predominance of CTX-M-15 and high genetic diversity. Microorganisms 2021; 9: 1914. 10.3390/microorganisms9091914 34576808 PMC8467980

[26] Nadimpalli ML, de Lauzanne A, Phe T et al *Escherichia coli* ST410 among humans and the environment in Southeast Asia. Int J Antimicrob Agents 2019; 54: 228–32. 10.1016/j.ijantimicag.2019.05.024 31176748

[27] Liao W, Liu Y, Zhang W. Virulence evolution, molecular mechanisms of resistance and prevalence of ST11 carbapenem-resistant *Klebsiella pneumoniae* in China: a review over the last 10 years. J Glob Antimicrob Resist 2020; 23: 174–80. 10.1016/j.jgar.2020.09.004 32971292

[28] Schmidt J, Zdarska V, Kolar M et al Analysis of BlaEC family class C beta-lactamase. FEMS Microbiol Lett 2023; 370: fnad097. 10.1093/femsle/fnad097 37757475 PMC10563145

[29] Chen C, Shi Q, Hu X et al Co-existence of KPC-2, LAP-2, and CTX-M-65 in an ST1469 multidrug-resistant *Klebsiella pneumoniae* strain in China. Infect Drug Resist 2022; 15: 6731–7. 10.2147/IDR.S392063 36444214 PMC9700460

[30] Noubam-Tchatat CC, Maurin E, Proust S et al MAST^®^ D72C test: a novel option for ESBL, AmpC and carbapenemase detection. Eur J Clin Microbiol Infect Dis 2024; 43: 1181–92. 10.1007/s10096-024-04829-4 38664291

